# Retrospective feasibility study of simultaneous integrated boost in cervical cancer using tomotherapy: the impact of organ motion and tumor regression

**DOI:** 10.1186/1748-717X-8-5

**Published:** 2013-01-03

**Authors:** Fernanda G Herrera, Sharon Callaway, Ela Delikgoz-Soykut, Mehtap Coskun, Laetitia Porta, Jean-Yves Meuwly, Joao Soares-Rodrigues, Leonie Heym, Raphael Moeckli, Mahmut Ozsahin

**Affiliations:** 1 Department of Radiation Oncology, Centre Hospitalier Universitaire Vaudois – CHUV, Rue du Bugnon 21, Lausanne, 1011, Switzerland; 2 Velocity Medical Solutions, Atlanta, USA; 3 Department of Radiology, Centre Hospitalier Universitaire Vaudois – CHUV, Lausanne, Switzerland

**Keywords:** Cervical cancer, IMRT-tomotherapy, Simultaneous integrated boost, SIB, Organ motion

## Abstract

**Background:**

Whole pelvis intensity modulated radiotherapy (IMRT) is increasingly being used to treat cervical cancer aiming to reduce side effects. Encouraged by this, some groups have proposed the use of simultaneous integrated boost (SIB) to target the tumor, either to get a higher tumoricidal effect or to replace brachytherapy. Nevertheless, physiological organ movement and rapid tumor regression throughout treatment might substantially reduce any benefit of this approach.

**Purpose:**

To evaluate the clinical target volume - simultaneous integrated boost (CTV-SIB) regression and motion during chemo-radiotherapy (CRT) for cervical cancer, and to monitor treatment progress dosimetrically and volumetrically to ensure treatment goals are met.

**Methods and materials:**

Ten patients treated with standard doses of CRT and brachytherapy were retrospectively re-planned using a helical Tomotherapy - SIB technique for the hypothetical scenario of this feasibility study. Target and organs at risk (OAR) were contoured on deformable fused planning-computed tomography and megavoltage computed tomography images. The CTV-SIB volume regression was determined. The center of mass (CM) was used to evaluate the degree of motion. The Dice’s similarity coefficient (DSC) was used to assess the spatial overlap of CTV-SIBs between scans. A cumulative dose-volume histogram modeled estimated delivered doses.

**Results:**

The CTV-SIB relative reduction was between 31 and 70%. The mean maximum CM change was 12.5, 9, and 3 mm in the superior-inferior, antero-posterior, and right-left dimensions, respectively. The CTV-SIB-DSC approached 1 in the first week of treatment, indicating almost perfect overlap. CTV-SIB-DSC regressed linearly during therapy, and by the end of treatment was 0.5, indicating 50% discordance. Two patients received less than 95% of the prescribed dose. Much higher doses to the OAR were observed. A multiple regression analysis showed a significant interaction between CTV-SIB reduction and OAR dose increase.

**Conclusions:**

The CTV-SIB had important regression and motion during CRT, receiving lower therapeutic doses than expected. The OAR had unpredictable shifts and received higher doses. The use of SIB without frequent adaptation of the treatment plan exposes cervical cancer patients to an unpredictable risk of under-dosing the target and/or overdosing adjacent critical structures. In that scenario, brachytherapy continues to be the gold standard approach.

## Introduction

The whole-pelvis irradiation technique for cervical cancer has evolved over the last decades with the introduction of intensity modulated radiotherapy (IMRT). In general, it is accepted that a dose between 45–50 Gy to the pelvis is the standard of care. Additional dose escalation is achieved by 3D image-guided conformal brachytherapy. Brachytherapy boost is the gold standard for women with cervical cancer, limiting the toxicity to the surrounding normal structures and achieving excellent tumor control rates. The brachytherapy dose distribution is also superior to external beam radiotherapy (EBRT) boost [1]. More recently, investigators have proposed the use of IMRT to treat cervical cancer with the aim of reducing toxicity, and some studies have reported a significant dose reduction to small bowel, bladder and rectum with a subsequent decrease in toxicity [2]. Encouraged by these studies, some groups have explored the possibility of administering a simultaneous integrated boost (SIB) to target cervical tumors, with the aim of delivering an accelerated treatment to gross disease [3-5]. The final purpose of this technique has been either dose escalation to get higher tumoricidal effect or a way to replace brachytherapy.

Cervical cancers are usually bulky, and evidence shows rapid tumor reduction over the course of chemo-radiotherapy (CRT), which raised concerns about missing the geographical target using IMRT techniques [6-8]. Furthermore, the current target volume of IMRT-SIB plans is based on a single image set taken at one point before the start of treatment. Therefore, the initial dosimetry and dose volume histograms (DVHs) might not necessarily represent the actual dose delivered to the tumor and organs at risk (OAR), which further adds to the uncertainties in the use of SIB. The purpose of this study is to evaluate the magnitude of the gross tumor volume (GTV) regression and the impact that this has on the clinical target volume-simultaneous integrated boost (CTV-SIB) motion during CRT. We report the dosimetric consequences of the anatomical changes after re-contouring the target and the OAR on each weekly megavoltage computed tomography (MV-CT) taken daily for set-up verification to ensure treatment goals are met.

## Materials and methods

Ten women with cervical cancer were retrospectively re-planned in a hypothetical scenario to study the feasibility of the SIB technique. Patients’ characteristics are described in Table 1. They had undergone standard whole pelvis irradiation using helical Tomotherapy (HT) with daily MV-CTs to a total dose of 50.4 Gy in 28 fractions of 1.8 Gy with concomitant weekly cisplatin chemotherapy. It is important to note that these women were all treated with magnetic resonance imaging (MRI)-guided intracavitary brachytherapy, and that the SIB technique was carried out only to evaluate the feasibility of the technique but was finally not implemented to treat these patients. The local ethical committee approved the study.

**Table 1:** Patients and tumor characteristics

**FIGO**	**N**
II A	3
IIB	5
IIIB	2
**Histology**	
Squamous	9
Adenocarcinoma	1
**Grade**	
2	5
3	5
**Lymph node Status**	
Pelvic positive	4
Para-aortic positive	1
Pelvic and para-aortic positive	4
Negative Lymph nodes	1
**Myometrial infiltration**	7
**Median tumor size 5 cm (range 4.5 to 8 cm)**	

### Imaging

Each patient underwent a pelvic CT scan (planning-CT) and MRI before starting CRT. In an attempt to minimize organ motion and as per standard practice in our department, patients received written instructions to use a mild laxative 48 h before the planning-CT and to drink 400 ml of water one hour before the CT after voiding completely. Patients were also advised to follow the same instructions during treatment. For the planning-CT, the acquisition parameters were as follows: tension 120 kV, tube rotation time 1 second, tube current 160 mAs, helical acquisition with pitch of 0.938, reconstructed image thickness 2 mm. Patients received intravenous contrast media.

As part of the daily treatment, on-board MV-CTs were obtained prior to each RT session using HT. The images were evaluated on-line by the radiation therapist, and if soft-tissue deviations were identified (due to variations in bladder or rectal filling), patients were taken off the treatment and the attending radiation oncologist was called to advise the patient on how to correct the physiological deviations of the OAR. Patients were then treated that same day with a full bladder and an empty rectum. A new MV-CT was then taken to ensure the correct set-up. In this latter situation, the latest correct MV-CT (full bladder – empty rectum) was used for this study. All MV-CTs were imported to Velocity Advanced Imaging Software (Velocity Medical Solutions, Atlanta, GA) and deformable fused with the planning-CT. Because of the retrospective nature of the study the patients did not have an MRI during the course of CRT.

### Delineation of clinical target volume (CTV) and planning target volume (PTV)

For accuracy on contouring, the T2-weighted MRI image sets at diagnosis were rigidly fused to the baseline planning-CT images using Velocity Advanced Imaging Software. The target and the OAR were contoured on the planning-CT and on each weekly MV-CT. All targets and OAR were contoured by a radiation oncologist (EDS) before being reviewed by a second investigator (FH), both with an experience of seven years in treating gynecological malignancies. In case of doubt or disagreement, a radiologist with ten years experience in gynecological cancer (JYM) provided precision in defining the anatomical structures.

**Figure 1:**
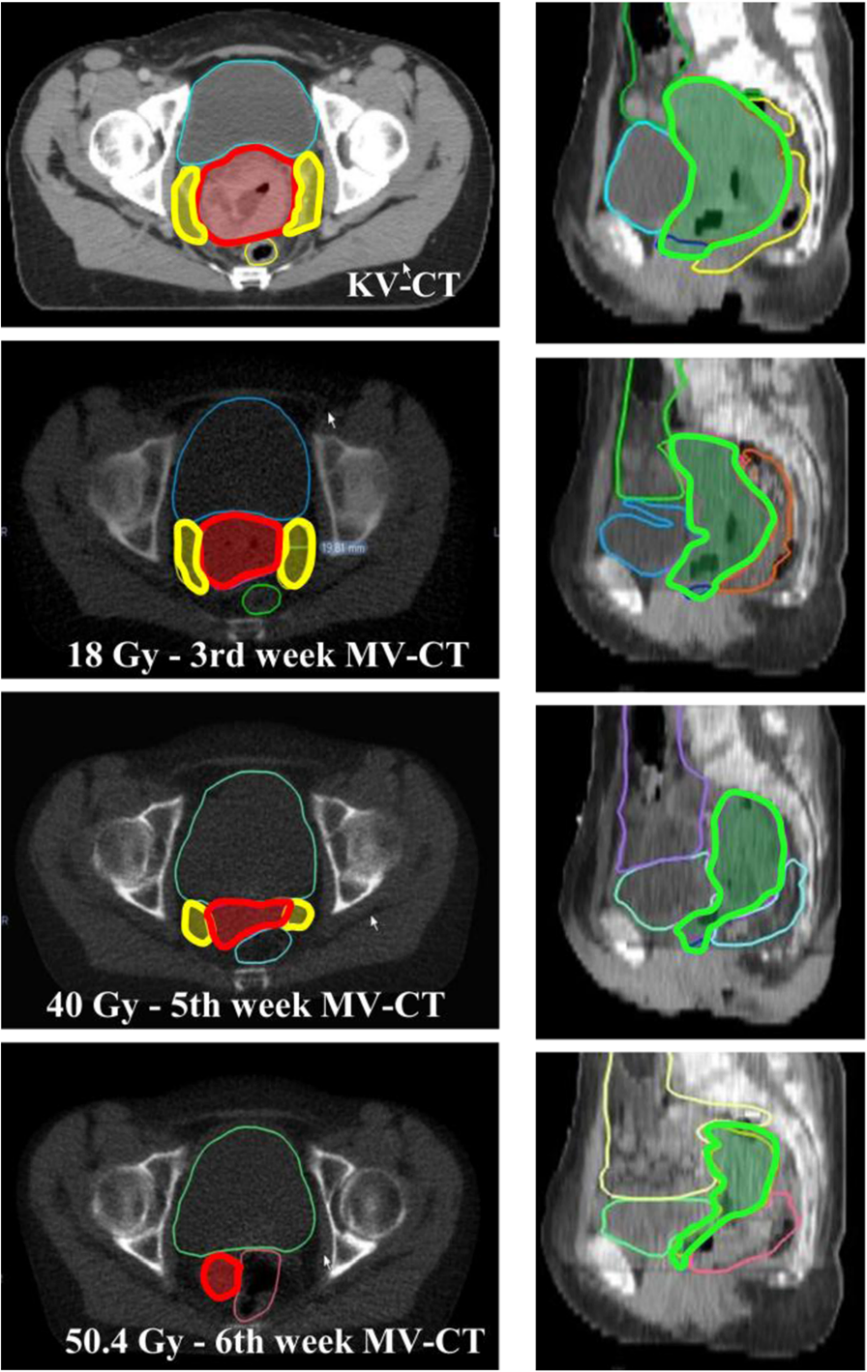
**Standardized guidelines on contouring the clinical target volume - simultaneous integrated boost (CTV-SIB) on the planning-CT and weekly MV-CTs.** Guidelines were adapted from Viswanathan et al. Axial and sagittal view of weekly contouring in one of the patients. The CTV-SIB structures included were: *In red*: The cervix containing the tumor. *In yellow*: Parametriums were divided as: · If inner half invasion: Butterfly shape structure <2 cm from cervix edge. · If outer half invasion: Butterfly shape structure > 2 cm from cervix edge. *In green (sagittal view)*: Corpus uteri. · If there was myometrial infiltration the entire corpus uteri was included. · If there was no myometrial infiltration, a 2-cm expansion was added from the proximal extend of the superior cervix edge or to a point at which volume expands (indicating presence of uterine tissue – where the uterine cavity appears). The vagina was contoured as a 2 cm expansion inferior to cervix edge.

The IMRT consortium guidelines were used to contour the pelvic CTV [9]. According to the same guidelines, a 1.5-cm uniform margin was added around the CTV to obtain the pelvic planning target volume (pPTV). The nodal CTV (nCTV) was delineated according to published guidelines [10]. A 7-mm uniform margin was added to the nCTV to obtain the nodal PTV (nPTV) [11]. No consensus exists on the structures to be included in the CTV-SIB. We encountered important limitations when contouring structures on MV-CT imaging due to low soft-tissue contrast. To overcome these limitations we used previously published guidelines on CT-based contouring for brachytherapy in cervical cancer [12]. Figure 1 describes the structures included and shows the contour delineation in one of the patients. A 1-cm uniform margin was added to the CTV-SIB to obtain the PTV-SIB. We chose this margin knowing from previous publications that a margin smaller than 1-cm might not achieve good target coverage, whilst with a larger margin the constraints of the OAR would not be respected [13].

### Delineation of OAR

The OAR were contoured on the fused planning-CT/MRI image sets and then re-contoured for comparisons on each weekly MV-CT. The delineated OAR were bladder, recto-sigmoid up to the sigmoid loop to the level of sacrum 1–2, and the small bowel as a whole peritoneal cavity or space that individual loops could occupy during treatment. No margin for planning organs at risk volume was applied.

### RT Planning

The prescribed dose to pPTV was 50.4 Gy in 28 fractions of 1.8 Gy. The dose prescribed to PTV-SIB was 59.36 Gy delivered in 2.12 Gy per fraction in 28 fractions. We did not increase the dose to the PTV-SIB to more than 60 Gy because this would have had a detrimental effect on the OAR.

The primary target coverage objectives were: 98% of CTV-SIB to be covered by 98% of the prescribed dose, and 98% PTV-SIB to be covered by 95% of the prescribed dose. Dose objectives for the OAR were based on the RTOG 0415 protocol, and were kept as low as possible without compromising PTV coverage. As the nodal volume is relatively fixed to bone, the nCTV was assumed not to change during the course of treatment, receiving its planned dose, and is not assessed in this study. The IMRT plans were performed on the Tomotherapy TPS (TomoTherapy Inc., Madison, WI) with a field width of 5 cm, and a pitch of 0.287.

### Dose accumulation

Dosimetric changes can occur due to either external anatomical changes or internal organ motion. In this study, our aim was to put into perspective the organ motion without considering the external anatomical changes. Therefore, we selected patients who did not have a significant change in their outer anatomy due to weight change and we assumed that external geometry did not change. Following that assumption, the dose distributions obtained from the planning-CT were fused to each weekly MV-CT using a deformable registration algorithm, assuring that the deformable dose matrix was applied in the same voxel position.

The total dose received by the CTV-SIB and the OAR over the course of treatment was calculated by plotting the cumulative DVH for each patient structure.

### Volumetric geometrical and positional analyses

To determine the displacements of the CTV-SIB during treatment, the Velocity software was used to place a geometric center of mass (CM) on each CTV-SIB. The coordinates of the CTV-SIB-CM on the planning-CT were then compared with the coordinates of all other CTV-SIB geometric centers for each weekly MV-CT. We determined the maximal displacements in right-left (x), anterior-posterior (y) and superior-inferior (z) directions. The maximum CTV-SIB displacements were measured for each patient, and the mean maximum CTV-SIB - displacements were calculated for the entire cohort. We also performed the same analysis for the OAR.

For each patient the spatial overlap between any two CTV-SIB contours was calculated using the Dice’s similarity coefficient (DSC) [14]. When the CTV-SIB structures on the planning-CT overlap perfectly with those on the MV-CTs, the DSC is 1. Complete discordance gives a value of 0. Values of DSC less than 0.5 indicate discordance exceeding 50%.

### Statistics

Multivariate analysis of variance (MANOVA) was used to compute the statistical differences in volume changes during treatment. The dosimetric consequences of volume change after weekly re-contouring were assessed by comparing the planned vs. delivered doses using a paired Student’s t-test.

A multiple regression analysis was used to determine which movement/volume factors contributed the most to the dose received to a given structure.

## Results

### CTV-SIB regression and motion

**Figure 2:**
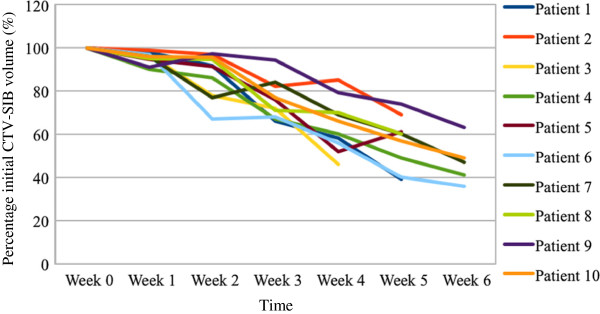
**Changes in the clinical target volume - simultaneous integrated boost (CTV-SIB) for each patient during the course of chemo-radiotherapy (CRT).** The volume of the CTV-SIB is plotted as a percentage of the CTV-SIB from the start of CRT. Percentages are plotted against time in weeks. Week 0 corresponds to measurements taken on the planning-CT before the start of CRT. Week 1–6 are measurements from the weekly MV-CTs.

The weekly MV-CTs showed important regression of the CTV-SIB during the course of CRT. The mean CTV-SIB was 237.38 cc (range 69.3 to 518) on the planning-CT and 112.55 cc (range 26.8 to 199.2) on the MV-CT at the end of CRT. The relative reduction in the CTV-SIB from baseline to the end of CRT was 31-70%. (MANOVA, p = 0.0002). (Figure 2) The median number of MV-CTs (correct after set-up verification) obtained per patient was 6 (range 4 to 6 scans).

**Figure 3:**
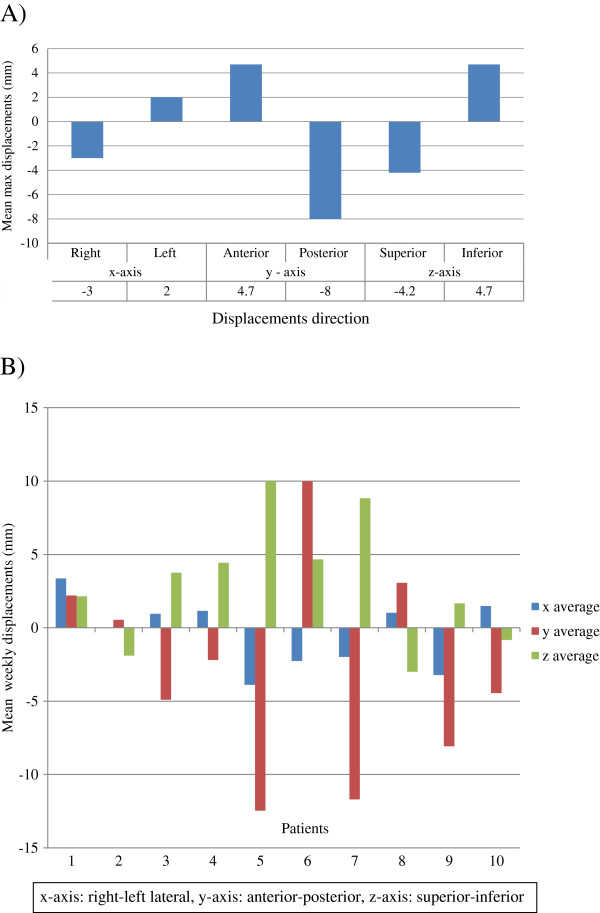
**Clinical target volume - simultaneous integrated boost (CTV-SIB) – center of mass (CM) displacements in millimeters (mm) from the original planning-CT center of mass position.A**) Mean maximal displacements of the CTV-SIB-CM in each dimension. **B**) Mean weekly CM displacements in individual patients.

The mean maximum change in CTV-SIB-CM position for the entire cohort was 4.7 mm anteriorly (range 0 to 13.4), -8 mm posteriorly (range −0.6 to −15.6), -4.2 mm superiorly (range −1.4 to −15), 4.7 mm inferiorly (range 0 to 15.5), -3 mm right-laterally (range −0.4 to −9.7), and 2 mm left-laterally (range 0 to 7.8) (Figure 3A).

Examining the individual patients represented in Figure 3B, we observe the intricate variation of the CTV-SIB-CM with mean weekly displacements up to 13 mm, 10 mm, and 4 mm in the anterior-posterior, superior-inferior, and right-left lateral axes, respectively.

**Figure 4:**
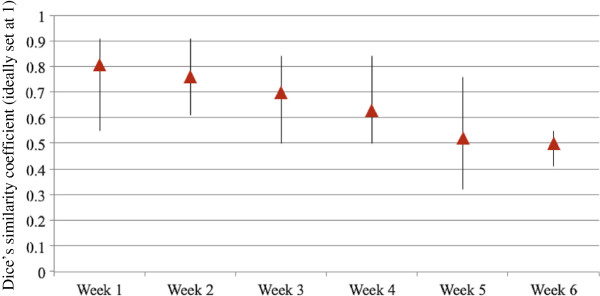
**Mean weekly Dice’s similarity coefficient (DSC) for the entire cohort.** Triangles represent mean values; the extensions of the lines represent range fluctuations.

The mean weekly DSC for the entire cohort showed a linear regression during the course of CRT (Figure 4). Interestingly, the mean weekly DSC during the first week of treatment was 0.8 (range 0.55 to 0.91) indicating a mean discordance of 20% between any two CTV-SIB contours under comparison. In contrast, at the end of CRT the mean weekly reduction in the DSC for the entire cohort was 0.5 (range 0.41 to 0.55), indicating more than 50% discordance, (MANOVA, p < 0.0001).

### OAR volume changes

Despite bowel and bladder preparation, important variation occurred during treatment. The maximal relative changes in bladder, recto-sigmoid, and bowel volume compared to baseline were 34 to 91%, 8 to 73.8%, 5 to 52%, respectively.

Significant changes of volume were seen over time. Bladder volume decreased significantly (MANOVA, p = 0.0001). The volume of recto-sigmoid and bowel increased significantly during the treatment period (MANOVA, p < 0.0001).

### Planned vs. delivered (accumulated) dose to CTV-SIB

In the patient cohort, the mean accumulated CTV-SIB dose to 98% volume (D98) decreased from 59.74 Gy to 58.89 Gy, (p = 0.54). However, in two individual patients, the CTV-SIB failed to meet the minimum acceptable 95% dose criteria. (Additional file 1: Appendix I-A). Beginning at the second week these two patients had a reduction in the CTV-SIB. As treatment progressed, the uterus became retroverted favoring the posterior movement of the target and resulting in a portion of CTV-SIB (isthmus, cervix and upper vagina) being outside of the PTV-SIB. These regions of 20 and 37 cc would have been under-dosed during most of the treatment with an accumulated D98 of 48.26 Gy and 43.13 Gy, respectively. (Additional file 2: Appendix II).

A multiple regression analysis (Table 2) shows the significant inter-relation between organ movement and CTV-SIB under-dosage. When the rectum moves anteriorly or reduces in volume the CTV-SIB receives a significantly lower dose (p = 0.029 and p = 0.01, respectively) When the CTV-SIB reduces in volume the DSC goes significantly down (p = 0.005).

**Table 2:** Multiple regression analysis

***CTV under-dosage***	***p-value***
Rectum moved anteriorly	0.029
Rectum reduced in volume	0.01
Reduction in the Dice’s coefficient	0.005
***Bowel doses increase***	***p-value***
Bladder moved anteriorly	0.01
Bladder reduced in volume	0.0046
***Rectal doses increase***	***p-value***
Rectal volume increased	0.0026
CTV-SIB moved anteriorly	0.00001
***Bladder doses increase***	***p-value***
Bladder moved posteriorly	0.01
CTV-SIB reduced in volume	0.01

Independent factors contributing to the clinical target volume - simultaneous integrated boost (CTV-SIB) under-dosage and organs at risk (OAR) over-dosage.

### Planned vs. delivered (accumulated) doses to OAR

Looking at the entire cohort, the planned and delivered (accumulated) doses to recto-sigmoid, bladder and bowel were not statistically different when compared to the original planning scenario (p = 0.96, p = 0.78, and p = 0.23, respectively). However, when we measure the OAR volumes exposed to more than 90% of the prescribed dose, 59.36 Gy (V53.4), individual patients had an increase in this volume. (Additional file 1: Appendix I-B). Taking all patients as a group, the V53.4 for bladder and bowel increased from 41.6% to 50.7% (p = 0.28), and from 7% to 14% (p = 0.01), respectively.

A multiple regression analysis (Table 2) shows the significant interplay between organ motion and CTV-SIB movements. When the bladder reduces in volume or moves anteriorly, the dose to the bowel increases significantly (p=0.0046, and p=0.01, respectively). When the CTV-SIB moves anteriorly, creating more space for the recto-sigmoid, the rectal dose goes up (p < 0.00001). In the same way, the bladder dose increases when the CTV-SIB reduces in volume (p=0.01).

## Discussion

IMRT has been shown to reduce treatment-induced toxicity but its use in cervical cancer has been limited because of significant organ movement [6]. Nevertheless, prompted by technological advances, some groups have speculated that SIB can replace the use of brachytherapy or eventually increase tumoricidal doses to the target [3,5,15]. Recent publications, mainly focused on optimization analysis, have proposed the use of SIB to treat cervical cancer [4]. However, the impact of organ motion on target coverage was not evaluated. Therefore, our study provides comprehensive information about the movement of the target and the OAR when SIB is used, with special focus on the dosimetrical consequences of this movement in women who underwent repetitive images during treatment.

We showed that the site and volume of the CTV-SIB changes significantly during CRT. These results are consistent with findings from other groups documenting a pelvic CTV relative reduction between 7-80% from baseline to the end of treatment due to tumor shrinkage [8]. Van de Bunt et al. observed rapid tumor regression using MRI scans before and after 30 Gy with a mean reduction in cervix tumor volume of 79% after three weeks of therapy [16]. Our results demonstrated a large variation of the CTV-SIB-CM, in the order of centimeters in some cases, with an important impact on the DSC. This is explained by geometrical changes in the position of the cervix and corpus uteri as well as variations in bladder and rectal filling. Chan et al. studied the internal movement of the tumor, cervix, and uterus using weekly cine-MRIs and a point of interest analysis (POI). The fundus POI drifted 1.5 cm caudally during CRT, and the cervical canal 1 cm. [7] Lee et al. used a metallic ring that was part of a brachytherapy uterine sleeve as a surrogate for cervix position. They reported motion in the medio-lateral, antero-posterior, and supero-inferior directions of 10, 16, and 8 mm, respectively [17]. This motion is in line with that observed in our study. However, it is important to recognize that the results of our study depend also on the CTV-SIB definition that we used and might not translate to other situations. It is important to note that in most of our patients a large area of corpus uteri was incorporated into the CTV-SIB. This part of the uterus is the most mobile one and might have contributed to an increase in the CM movement as well as a decrease in the DSC. Nevertheless, our CTV-SIB definition is consistent with recent published guidelines of brachytherapy [18,19]. The use of fiducial markers has also been proposed to define the SIB in cervical cancer [4]. However, systematic and random displacements of the markers relative to bony anatomy have been reported to be up to 1 cm [20].

We observed two patients with important target-missing with parts of the CTV-SIB moving outside the PTV-SIB. These patients would have needed posterior PTV-SIB margins of at least 1.5 cm, which would have been detrimental for the OAR, especially in the context of relatively high doses per fraction. The use of daily imaging would not have compensated for the magnitude of the movement, and periodic revisions of the treatment plan would have been necessary (adaptive re-planning). The dosimetric consequences of this target-missing might not be significant as some portions of the CTV-SIB continued to receive at least some dose, and remained well contained in the pelvis PTV. This might explain why previous studies of dose escalation with SIB and brachytherapy boost have so far reported optimal local control rates [4,21]. Nevertheless, target-missing with SIB can be clinically important if clinicians decide to apply lower doses of brachytherapy or simply if brachytherapy is withheld, which might expose patients to the catastrophic consequences of a local recurrence. Recent reports where radiation at doses of 54–70 Gy was applied using 3D-EBRT techniques, in patients regarded as inappropriate for brachytherapy, indicated an excessive local failure rate of 48% [22].

For this feasibility study, a relatively conservative SIB dose was modeled (from 50.4 to 59.4 Gy). This dose was chosen considering the large volume of PTV-SIB and the total amount of healthy tissue exposed. It has been previously published that large volumes in the pelvic region receiving more than 60 Gy correlates with increased pelvic side effects [23]. The SIB dose is another unresolved issue that should be considered before implementing this technique to treat cervical cancer. It must be remembered that squamous cell carcinoma of the cervix shows dose dependency with a greater response and local control with higher doses of radiation [24]. The use of relatively low doses per fraction with SIB might eventually compromise outcome. Conversely, in brachytherapy, the dose to the GTV is higher owing to the vicinity of the sources, and the doses to the OAR have a steep dose gradient. In brachytherapy, target coverage is not compromised as individual MRI-based plans are performed before each intracavitary insertion [25]. Furthermore, an IMRT-SIB boost with lower doses per fraction compared to brachytherapy should not compromise the total duration of treatment time which has been found to have a significant effect on pelvic control. Fyles et al. found that in 830 patients with cervical cancer treated by radical radiation therapy the loss of control was 1% per day of treatment prolongation beyond 30 days [26]. In addition, important concepts such as the need for generous margins make IMRT-SIB not comparable to brachytherapy. Even though, IMRT has challenged brachytherapy in different studies [27]. For example, tomotherapy boost was compared with a simple brachytherapy technique based on a Fletcher-Suit applicator [28]. Molla et al. reported on 15 patients who received IMRT boost as an alternative to brachytherapy [29]. The conclusions drawn from these studies were that IMRT allowed better OAR-sparing and more homogeneous dose distributions. However, these conclusions were biased because IMRT was compared with old brachytherapy techniques. Certainly, brachytherapy is much less homogeneous as the highest dose volume (>200% of the dose) is located within the applicator, and directly in the GTV. These high doses delivered in the central part of the tumor are the key for the success of this treatment modality. More recent advances attempted a comparison between high-tech EBRT and MRI-guided brachytherapy in locally advanced cervical cancer [30]. Georg et al. compared IMRT with photons and protons to deliver the highest possible dose to the PTV while respecting the dose to 1 cc (D_1cc_) and 2 cc (D_2cc_) limits of brachytherapy using the same fractionation (4 fractions of 7 Gy). Nine patients treated with intracavitary or interstitial brachytherapy were selected and re-planned using 3 and 5 mm margins around the high-risk and intermediate-risk CTV to construct the external beam-PTV. In this study, when the brachytherapy dose constraints were applied (D_2cc_ and D_1cc_) to the IMRT plans, the minimal dose that covered 90% of the high-risk and intermediate-risk external beam-PTVs was lower for the IMRT-plans. On the other hand, volumes receiving 60 Gy were approximately twice as large for IMRT compared to brachytherapy [1]. In summary, these studies showed that for cervical cancer boost treatments, IMRT is inferior to modern brachytherapy.

In the context of significant intra-patient variability with complex tumor and organ dynamics, we believe there is a place for improvement through the use of adaptive planning strategies using deformable registration. In the series of the Princess Margaret Hospital, 33 cervical cancer patients were evaluated with a 3 mm PTV margin with and without weekly adaptive re-planning [8,31]. In the non-adaptive scenario, 27% patients failed acceptable target coverage; this percentage was reduced to 3% when an adaptive strategy was applied with significant sparing to the OAR. In this regard, our dose accumulation relies upon perfect external anatomy configuration. This ideal scenario might be very different from real practice where the dose delivered to the tumor and the OAR might differ from the planned one because the patient’s geometry and internal anatomy varies over the course of treatment. This major technological challenge implies the use of deformable registration algorithms combined with adaptive re-planning. Nevertheless, we think that using accumulated doses derived from our deformable registration algorithm was the most suitable methodology to evaluate volumetric and dosimetric uncertainties, without taking into account the changes in the patients’ external geometry.

An important question that we should be able to answer in the near future is whether image-guided adaptive external beam radiotherapy (IGART) can be combined with image-guided adaptive brachytherapy (IGAB). With this approach, IGAB could utilize the dose reduction in the OAR achieved by IGART, in order to further improve the therapeutic ratio and to deliver a personalized radiation dose to each patient with an opportunity for dose escalation. For that purpose composite IMRT and brachytherapy plans should be fully integrated, and voxel by voxel tracking from both techniques could provide an advantage in the treatment of these women.

This study has several limitations. First, we retrospectively evaluated the feasibility of SIB in cervical cancer using weekly MV-CT images rather than MRI to contour cervical boundaries. The MV-CTs are part of the daily images taken for set-up verification with HT and the study was done in this way for practical reasons. The use of MV-CTs might have led to overestimation of the size of the CTV-SIB, overshadowing the already significant results. Previous work on cervical cancer patients has shown that MV-CT guidance allows the identification and contouring of the cervix with minimal intra- and inter-observer variability among the investigators [32]. In addition, the data obtained from our weekly MV-CTs were extrapolated to the whole course of treatment, which might have produced an overestimation of systematic errors. The intra-fraction motion of the target was assumed to be zero which might have further under-estimated random errors. The set-up errors were assumed to be negligible because of daily online MV-CT corrections.

## Conclusions

The structures contained in the CTV-SIB show a significant regression during the course of CRT. Uterus and OAR change in volume, shape and location along the course of treatment. These unpredictable changes make the delivery of IMRT and particularly SIB challenging. The use of daily MV-CT imaging and considerable PTV margins in the absence of an adaptive strategy could not compensate for the CTV-SIB positional changes with therapeutic doses lower than expected and higher doses to the OAR. In this context, it is nearly impossible to perform an SIB with a dose comparable to brachytherapy. SIB has to assure tumor control without significantly increasing the irradiation to normal tissues, and using adaptive planning strategies coupled with deformable registration are expected to add a significant improvement in the delivery of IMRT-SIB. However, in the absence of deformable registration and adaptive re-planning, the use of IMRT-SIB should be avoided. Brachytherapy continues to be the gold standard.

## Competing interests

Sharon Callaway is an employee of Velocity Medical Solutions. She does not receive royalties derived from Velocity Medical Solution’s sale of products. The authors declare that they have no competing interests.

## Authors’ information

Oral presentation at the 31st Annual Meeting of the European Society for Radiotherapy and Oncology (ESTRO), Barcelona 9-10th May, 2012. Abstract number: 0126.

## Authors’ contributions

FH: carried out the conception and design of the study, provided study patients, participated in data analysis and interpretation, and drafted the manuscript. SC: participated in the collection and assembly of data, data analysis and interpretation. EDS: participated in the contouring part of the study. MC: contributed to the conception of the study and participated in the contouring part. LP: participated in data analysis and interpretation. JYM: provided expert support in image evaluation and contouring. JSR: participated in the re-planning part of the study. LH: participated in the re-planning part of the study. RM: contributed to the conception of the study, participated in data analysis. MO: contributed to the conception and design of the study, participated in data analysis and interpretation, collaborated in drafting the manuscript. All authors read and approved the final manuscript.

## Supplementary Material

Additional file 1 **Appendix 1.** A)Histograms of planned vs. accumulated delivered doses to clinical target volume – simultaneous integrated boost (CTV-SIB) for each patient in the study. ICRU 50 specifies the acceptable dose range as 95–107% of the prescribed dose. The arrows indicate patients who received less than 95% of the prescribed dose. B)Histograms of planned vs. accumulated delivered doses to the OAR in individual patients. In the y-axis is the tissue volume receiving more than 90% of the prescribed dose, 59.36 Gy (V53.4). Click here for file

Additional file 2 **Appendix 2.** Target motion: Axial and sagittal view in one patient. In both figures the blue contour is the pre-treatment clinical target volume-simultaneous integrated boost (CTV-SIB) with a 1-cm margin planning target volume (PTV-SIB) black contour encompassed by the 95% isodose curve. For this patient the PTV-SIB does not cover a posterior shift of the CTV-SIB from week 2 until the end of treatment (lighter contours). In the MATLAB graphic we observe the three dimensional movement of the vectors in this same patient. Click here for file
