# Supplementary material for: Retrospective feasibility study of simultaneous integrated boost in cervical cancer using tomotherapy: the impact of organ motion and tumor regression
Source: Radiat Oncol. 2013 Jan 3;8:5. doi: 10.1186/1748-717X-8-5 (PMC3551799; doi:10.1186/1748-717X-8-5)
Supplement: Additional file 1 — Appendix 1. A)Histograms of planned vs. accumulated delivered doses to clinical target volume – simultaneous integrated boost (CTV-SIB) for each patient in the study. ICRU 50 specifies the acceptable dose range as 95–107% of the prescribed dose. The arrows indicate patients who received less than 95% of the prescribed dose. B)Histograms of planned vs. accumulated delivered doses to the OAR in individual patients. In the y-axis is the tissue volume receiving more than 90% of the prescribed dose, 59.36 Gy (V53.4). [file 1748-717X-8-5-S1.doc]

**Appendix I.**

1. Histograms of planned vs. accumulated delivered doses to clinical target volume – simultaneous integrated boost (CTV-SIB) for each patient in the study. ICRU 50 specifies the acceptable dose range as 95–107% of the prescribed
   dose. The arrows indicate patients who received less than 95% of the prescribed dose.


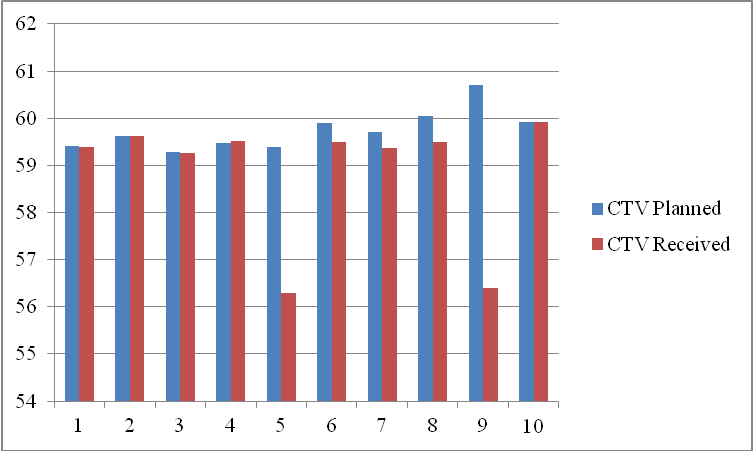


Dose to CTV-SIB (Gy)

Patients

1. Histograms of planned vs. accumulated delivered doses to the OAR in individual patients. In the y-axis is the tissue volume receiving more than 90% of the prescribed dose, 59.36 Gy (V53.4).


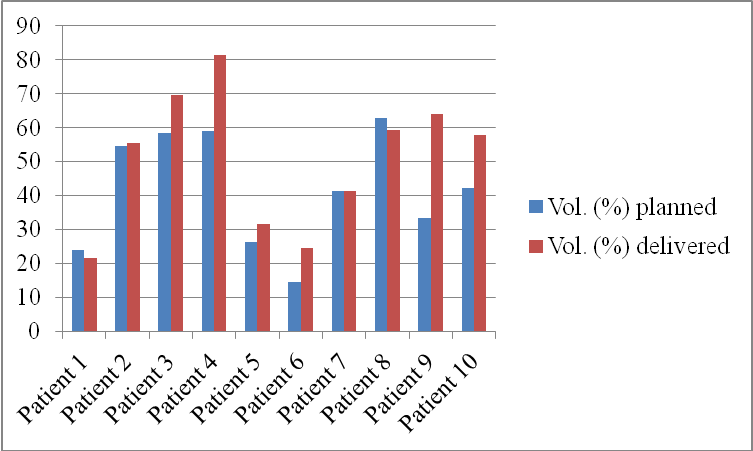


Volume (cc)

Bladder


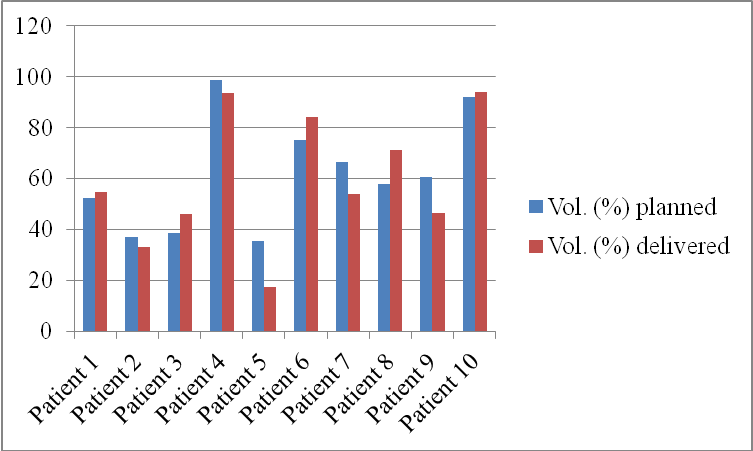


Volume (cc)

Recto-sigmoid


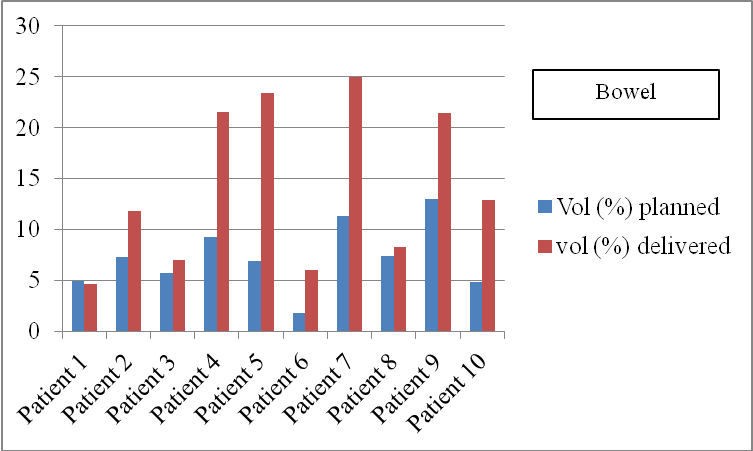


Volume (cc)
