# Supplementary material for: Retrospective feasibility study of simultaneous integrated boost in cervical cancer using tomotherapy: the impact of organ motion and tumor regression
Source: Radiat Oncol. 2013 Jan 3;8:5. doi: 10.1186/1748-717X-8-5 (PMC3551799; doi:10.1186/1748-717X-8-5)

Appendix II

Target motion: Axial and sagittal view in one patient. In both figures the blue contour is the pre-treatment clinical target volume-simultaneous integrated boost (CTV-SIB) with a 1-cm margin planning target volume (PTV-SIB) black contour encompassed by the 95% isodose curve. For this patient the PTV-SIB does not cover a posterior shift of the CTV-SIB from week 2 until the end of treatment (lighter contours). In the MATLAB graphic we observe the three dimensional movement of the vectors in this same patient.


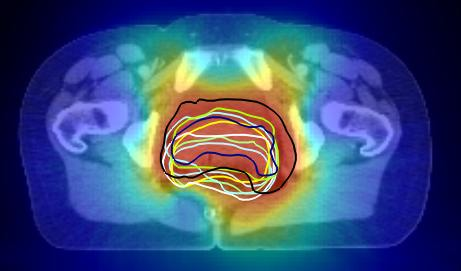


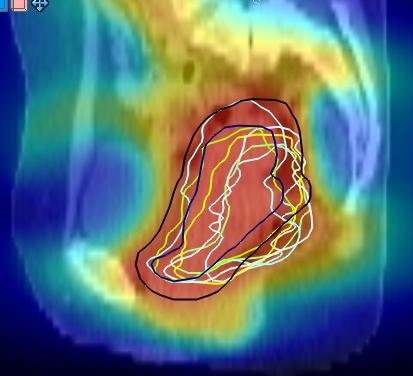


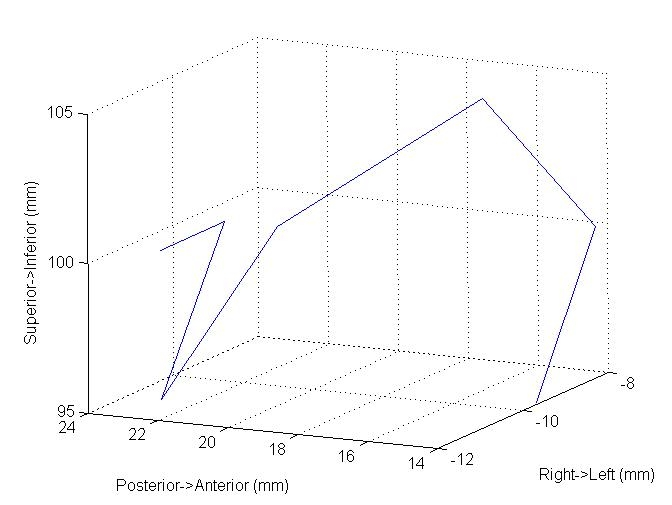

Supplement: Additional file 2 — Appendix 2. Target motion: Axial and sagittal view in one patient. In both figures the blue contour is the pre-treatment clinical target volume-simultaneous integrated boost (CTV-SIB) with a 1-cm margin planning target volume (PTV-SIB) black contour encompassed by the 95% isodose curve. For this patient the PTV-SIB does not cover a posterior shift of the CTV-SIB from week 2 until the end of treatment (lighter contours). In the MATLAB graphic we observe the three dimensional movement of the vectors in this same patient. [file 1748-717X-8-5-S2.doc]
